# Association between high triglyceride-glucose index and MACCE in hypertriglyceridemia patients undergoing percutaneous coronary intervention

**DOI:** 10.3389/fendo.2025.1519895

**Published:** 2025-04-14

**Authors:** Yichuan Wang, Yanfeng Lu, Shanshan Gao, Zhong Zhong, Jasmine Yimeng Bao, Bo Liu, Ruihan Fan, Ning Guo

**Affiliations:** ^1^ Department of Cardiovascular Medicine, The First Affiliated Hospital of Xi’an Jiaotong University, Xi’an, Shaanxi, China; ^2^ Sidney Kimmel Medical College, Thomas Jefferson University, Philadelphia, PA, United States

**Keywords:** triglyceride-glucose index, triglycerides, coronary artery disease, percutaneous coronary intervention, hypertriglyceridemia

## Abstract

**Background:**

With a focus on metabolism-related cardiovascular diseases, the triglyceride-glucose (TyG) index has been used as a surrogate marker of insulin resistance in the prognosis of coronary heart disease. However, the prognostic role of the TyG index in patients with elevated triglycerides, still requires further research. This study aimed to investigate the association between the TyG index and Major Adverse Cardiac and Cerebrovascular Events (MACCE) in patients with hypertriglyceridemia undergoing drug-eluting stent percutaneous coronary intervention (DES-PCI).

**Methods:**

Out of 2250 patients, 813 with hypertriglyceridemia who underwent DES-PCI were retrospectively analyzed. MACCE was regarded as the primary endpoint. Kaplan–Meier (KM) curves were used to evaluate the association between the TyG index and different endpoints. Restricted cubic spline (RCS) analysis was used to examine the relation between the TyG index and MACCE. Subgroup analysis was conducted to further evaluate the interaction between the TyG index and subgroup indicators.

**Results:**

Cox regression analysis identified the TyG index as an independent predictor of MACCE (hazard ratio [HR] 1.53, 95% confidence interval [CI] 1.15–2.04, P = 0.004). Receiver operating characteristic (ROC) analysis determined 9.19 as the cutoff value of TyG index. The Kaplan–Meier curve indicated that patients with a TyG index > 9.19 had higher risks of MACCE (HR 2.23, 95% CI 1.35–3.67, *P* = 0.002), MACE (HR 2.38, 95% CI 1.39–4.09, P = 0.002), unplanned repeat revascularization (HR 2.05, 95% CI 1.02–4.09, P = 0.043) and all-cause death (HR 3.31, 95%CI 1.15–9.47, P = 0.026) than those of patients with a low TyG index. RCS analysis revealed a linear relation between the TyG index and MACCE risk (P for nonlinearity = 0.879, P for overall trend = 0.044).

**Conclusions:**

This study demonstrated that a high TyG index is associated with an increased risk of MACCE, suggesting that the TyG index may serve as a valuable prognostic marker in patients with hypertriglyceridemia undergoing DES-PCI.

## Introduction

Atherosclerotic cardiovascular disease is the primary cause of chronic heart disease, resulting in continuous clinical and economic burdens. Although the low-density lipoprotein cholesterol (LDL-C) has been the primary target of treatment for decades, the effect of triglycerides (TGs) on residual lipoprotein risk remains controversial (1). Hypertriglyceridemia is characterized by an elevated plasma TG level exceeding 150 mg/dL (1.7 mmol/L). Mild-to-moderate hypertriglyceridemia is mainly associated with the development of atherosclerotic plaques, even leading to pancreatic dysfunction (2). The incidence of elevated TG levels and hypertriglyceridemia in the Chinese population has been rising annually (3). According to the DYSIS-China National Cross-Sectional Study, even after initiating treatment with cholesterol-lowering medications, such as statins, at least 40% of patients exhibit persistently elevated TG levels (4).

The TyG index has been found to correlate with many metabolism-related diseases, such as non-alcoholic fatty liver disease, stroke and kidney injury. Insulin resistance (IR), which involves impaired glucose absorption and utilization, plays an important role in the development of metabolic syndrome and cardiovascular disease (5). The hyper insulinemic-euglycemic clamp (HIEC) technique is the gold standard for quantifying IR. The HIEC technique uses the simultaneous infusion of exogenous insulin and glucose to increase plasma insulin levels while maintaining blood glucose at basal homeostasis levels to evaluate insulin sensitivity but is limited because of its cost and complexity. The homeostasis model assessment of IR (HOMA-IR) technique offers similar results to HIEC within a specific range. However, when islet cells are reduced or fail, accurate quantification of IR cannot be performed (6). The TyG index, a non-insulin-based marker, offers a more cost-effective alternative by combining fasting blood glucose and TG measurements (7). The TyG index has demonstrated a stronger association with diabetes in Korean individuals who were not underweight or obese than in those with HOMA-IR (8). A comparison of the TyG index, TG/high-density lipoprotein cholesterol (HDL-C) ratio levels in patients who underwent PCI at Fu Wai hospital showed that the TyG index is a valuable non-insulin-based parameter for metabolic evaluation in patients undergoing PCI (9). A largescale real-world study across five continents found that the TyG index was closely related to future cardiac-cerebral vascular diseases and diabetes (10). Despite these findings, the relation between the TyG index and hypertriglyceridemia requires further exploration. Our study aimed to investigate the relation between the TyG index and Major Adverse Cardiac and Cerebrovascular Events (MACCE) in patients with hypertriglyceridemia undergoing drug-eluting stent percutaneous coronary intervention (DES-PCI), addressing the gap in studies regarding the TyG index in lipid metabolism disorders. These findings may provide a valuable reference for lipid-lowering treatment post-DES-PCI and offer new evidence for the application of the TyG index in patients with coronary artery disease (CAD).

## Materials and methods

### Study population

**Figure 1:**
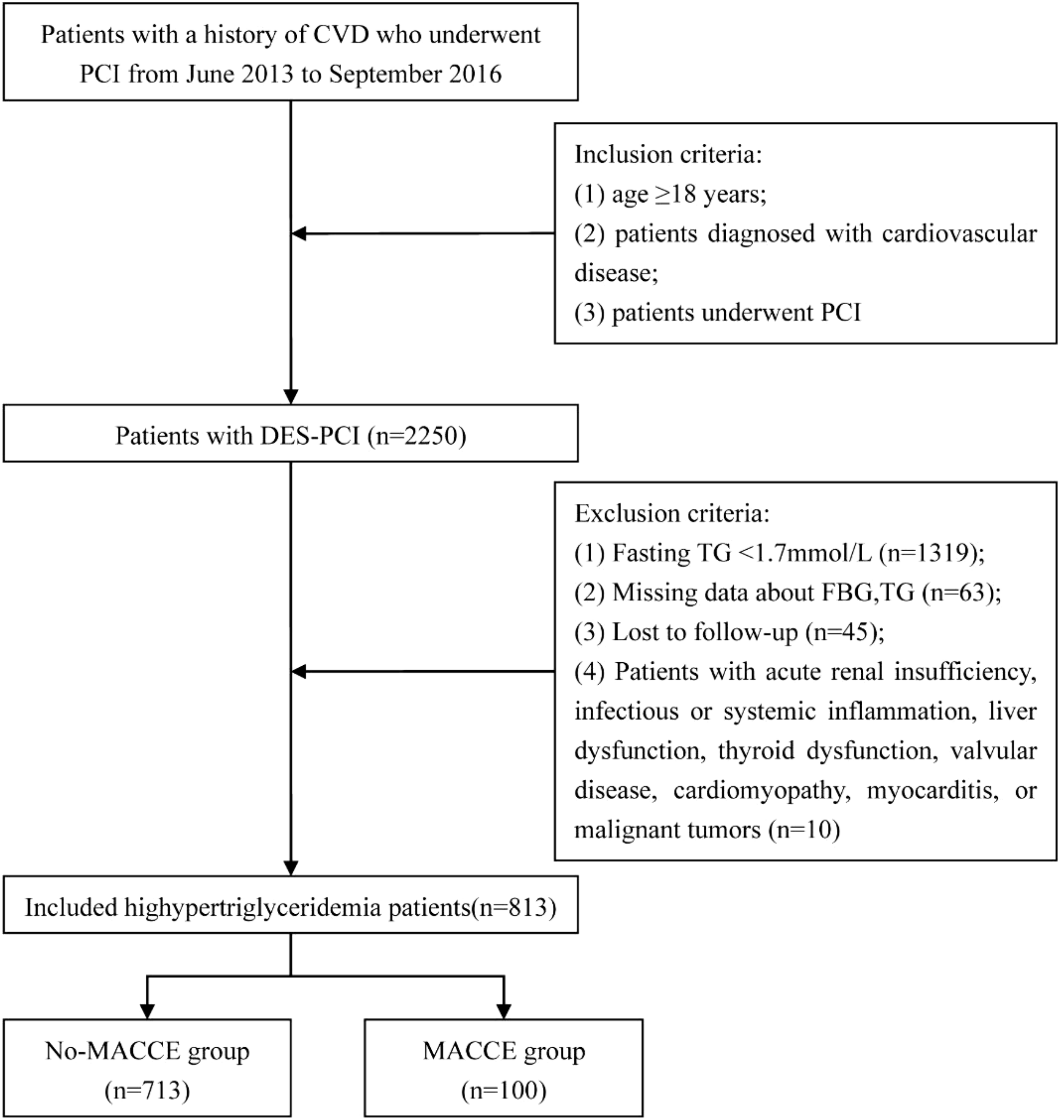
Enrollment flow chart. DES-PCI, drug-eluting stent percutaneous coronary intervention; FBG, fasting blood glucose; TG, triglyceride; MACCE, major adverse cardiac and cerebrovascular events; TyG, triglyceride-glucose index.

This was a single-center, retrospective, observational cohort study. Data on consecutive patients who underwent DES-PCI were obtained from the Hospital Information System Database at the First Hospital Affiliated of Xi’an Jiaotong University, between June 2013 and September 2016. Hypertriglyceridemia was defined as fasting plasma TG ≥ 1.7 mmol/L. The inclusion criteria were as follows: (1) age ≥ 18 years; (2) diagnosed with CAD; and (3) having undergone PCI and DES implantation. The exclusion criteria are shown in **Figure 1**. Of the 2250 patients, 813 were ultimately enrolled in the study after excluding patients with plasma TG levels < 1.7 mmol/L (n = 1319) and those who lacked lipid or glucose test results (n = 63). Patient lost to follow-up (n = 45) were also excluded. Patients were classified into two groups based on the occurrence of MACCE during the follow-up period: the MACCE (n = 100) and no-MACCE groups (n = 713).

### Data collection and definitions

Demographic characteristics, clinical history, family history of CAD, laboratory indicators, and angiographic data, were obtained from the electronic medical record system of the First Affiliated Hospital of Xi’an Jiaotong University. The demographic characteristics included age, sex, baseline blood pressure, and heart rate. Patient history included smoking, drinking, established type 2 diabetes mellitus (T2DM), hypertension, stroke, previous myocardial infarction, and dyslipidemia. T2DM was defined by the following criteria: FPG levels ≥ 7.0 mmol/L, random blood glucose levels ≥ 11.1 mmol/L, 2- h plasma glucose levels after an oral glucose tolerance test (OGTT) ≥ 11.1 mmol/L, or the utilization of insulin or oral hypoglycemic agents. Dyslipidemia was characterized using the ICD-10 code E78 along with the use of lipid-lowering medications or a total cholesterol level ≥ 240 mg/dL. Hypertension was defined as systolic blood pressure ≥ 140 mmHg and/or diastolic blood pressure ≥ 90 mmHg, or treatment with antihypertensive drugs. Laboratory tests included white blood cell count, platelets, red blood cell count, hemoglobin, total cholesterol, TG, LDL-C, HDL-C, serum creatinine, fasting blood glucose, and glycated hemoglobin A1c (HbA1c). These tests were performed according to standardized procedure and assay systems. Blood samples were collected before PCI. The TyG index was calculated as ln [fasting TG (mg/dL) × fasting glucose (mg/dL)/2].

### Percutaneous coronary intervention

DES-PCI, which refers to DES implantation into the target vessel, was performed by experienced operators following standard procedures. All patients received aspirin (300 mg), ticagrelor (180 mg), or clopidogrel (300 mg) before the procedure, and aspirin (100 mg QD), ticagrelor (90 mg BID), or clopidogrel (75 mg QD) after the procedure. Coronary angiography and PCI were performed according to standard protocols. Angiographic results were recorded by the cardiac catheterization laboratory, and every PCI report was reviewed by at least two independent cardiologists.

### Follow-up and endpoints

Follow-ups were conducted by doctors, either in outpatient settings or via telephone, during the follow-up period (every 30 d, then every 6 months for up to 6 years). The primary endpoint was a composite of the first occurrence of all MACCEs within the 66-month follow-up duration. The primary endpoint was MACCE, defined as follows: (1) all-cause death, (2) nonfatal myocardial infarction, (3) nonfatal stroke, and (4) unplanned repeat revascularization. The other outcomes were major adverse cardiovascular events (MACE), unplanned repeat revascularization, and all-cause death.

### Statistical analysis

Statistical analyses were performed using R version 4.2.1. All tests were two-tailed, with statistical significance set at a P-value < 0.05. Continuous variables are expressed as medians (interquartile range), whereas categorical variables are reported as frequencies and percentages. Receiver operating characteristic (ROC) curves were generated to compare the TyG index with fasting blood glucose, TG, LDL-C, pro–brain natriuretic peptide (pro-BNP), and HbA1c levels to predict MACCE in patients with hypertriglyceridemia. The Kaplan–Meier curve tracked survival based on different endpoints in patients with a high or low TyG index. Cox regression analysis demonstrated an independent association between the TyG index and MACCE occurrence. Subgroup analyses were conducted based on age, sex, hypertension, T2DM, multi-vessel disease, and laboratory measurements.

## Results

### Baseline characteristics

Ultimately, 813 participants were included in the study. The median follow-up period was 66 months (5.5 years). Among these patients, 100 (12.3%) experienced MACCE within 1 year whereas 713 did not (**Table 1**). The average age of the participants was 59 years, and 73.6% were men. Participants who experienced MACCE had high proportions of previous myocardial infraction, multivessel- CAD, and insulin use (all P < 0.05). Additionally, patients with MACCE had elevated levels of FPG, TG, BNP, and HbA1c, as well as significant differences in left ventricular ejection fraction (LVEF) and hemoglobin (all P < 0.05). Based on the cut-off value of the ROC analysis in **Supplementary Figure S1**, patients were divided into two groups: TyG < 9.19 and TyG > 9.19 (**Table 2**). Patients with a TyG index > 9.19 were more likely to have hypertension, T2DM, and dyslipidemia (P < 0.05). Regarding laboratory measurements, patients with a high TyG index tended to have high plasma TG, total cholesterol, FPG, HDL-C, and HbA1C levels(all P < 0.05).

**Table 1:** Baseline characteristics of population stratified by MACCE.

Characteristics	Total population (N=813)	No-MACCE (N=713)	MACCE (N=100)	P value
Demographics				
Age (years)	59.27 (51.87, 66.53)	59 (52, 66)	62 (55, 71)	0.018
Male (n, %)	598 (73.6%)	526 (73.8%)	72 (72%)	0.707
DBP (mmHg)	80 (70, 86)	80 (70.5, 86)	80 (70, 85)	0.326
SBP (mmHg)	130 (120, 145)	130 (120, 145)	131 (120, 147)	0.594
HR (bpm)	75 (68, 81)	75 (68, 80)	76 (68, 84)	0.197
Case history (n, %)				
Smoking	436 (53.6%)	381 (53.4%)	55 (55%)	0.769
Drinking	233 (28.7%)	209 (29.3%)	24 (24%)	0.271
T2DM	239 (29.4%)	200 (28.1%)	39 (39%)	0.024
Hypertension	507 (62.4%)	439 (61.6%)	68 (68%)	0.214
Previous MI	70 (8.6%)	56 (7.9%)	14 (14%)	0.040
Dyslipidemia	183 (22.5%)	164 (23.0%)	19 (19%)	0.370
Clinical diagnosis (n, %)				
UA	515 (63.3%)	449 (63.0%)	66 (66%)	0.556
NSTEMI	32 (3.9%)	30 (4.2%)	2 (2%)	0.430
STEMI	237 (29.2%)	212 (29.7%)	25 (25%)	0.329
Coronary angiography (n, %)				
Diffuse lesions	487 (59.9%)	422 (59.2%)	65 (65%)	0.267
Chronic total occlusions	227 (27.9%)	198 (27.8%)	29 (29%)	0.797
Multi-vessel disease	409 (50.3%)	346 (48.5%)	63 (63%)	0.007
Number of stents	2 (1,3)	2 (1,3)	2(1,3)	0.489
Medications at discharge (n, %)				
Aspirin	811 (99.8%)	712 (99.9%)	99 (99%)	0.584
Clopidogrel	726 (89.3%)	637 (89.3%)	89 (89%)	0.918
Ticagrelor	89 (10.9%)	76 (10.7%)	13 (13%)	0.483
ACEI/ARB	691 (85%)	602 (84.4%)	89 (89%)	0.231
β-blockers	670 (82.4%)	581 (81.5%)	89 (89%)	0.065
Insulin	71 (8.7%)	57 (8.0%)	14 (14%)	0.046
Laboratory measurements				
WBC (10^9/L)	6.93 (5.73, 8.57)	6.96 (5.74, 8.64)	6.64 (5.52, 8.20)	0.284
Hb (g/L)	144 (132, 153)	145 (132, 154)	141.5 (125.75, 151.25)	0.016
PLT (10^9/L)	200 (160, 238)	200 (160, 238)	198.5 (158.75, 236.5)	0.903
Creatinine (umol/L)	66 (56.38, 77.7)	65.5 (56.5, 77.1)	68.55 (55.87, 83.05)	0.081
TC (mmol/L)	4.24 (3.69, 4.82)	4.22 (3.69, 4.78)	4.46 (3.67, 5.05)	0.200
TG (mmol/L)	2.27 (1.93, 2.98)	2.24 (1.92, 2.95)	2.6 (2.05, 3.14)	0.011
FPG (mmol/L)	6.09 (4.93, 8.25)	5.95 (4.91, 8.15)	6.715 (5.33, 9.11)	0.019
TyG index	9.40 (9.09, 9.78)	9.38 (9.07, 9.75)	9.48 (9.23, 9.95)	0.007
LDL-C (mmol/L)	2.49 (2.01, 2.99)	2.48 (2.02, 2.98)	2.59 (1.96, 3.17)	0.475
HDL-C (mmol/L)	0.9 (0.77, 1.04)	0.9 (0.77, 1.04)	0.9 (0.77, 1.06)	0.890
Pro-BNP (ng/L)	193.9 (72.74, 600.62)	187.6 (67.9, 572.3)	283.3 (85.88, 862.55)	0.025
HbA1C (%)	5.8 (5.4, 6.78)	5.75 (5.4, 6.6)	6.15 (5.68, 7.43)	0.001
LVEF (%)	64 (56, 69)	65 (57, 69)	62 (48, 68)	0.011

DBP, diastolic blood pressure; SBP, systolic blood pressure; T2DM, type 2 diabetes mellitus; MI, myocardial infarction; PCI, percutaneous coronary intervention; UA, unstable angina; NSTEMI, non-ST-segment elevation myocardial infarction; STEMI, ST-segment elevation myocardial infarction; ACEI/ARB, angiotensin converting enzyme inhibitor or angiotensin receptor blocker; WBC, white blood cell; Hb, hemoglobin; PLT, platelet; TG, triglyceride; TC, total cholesterol; FPG, fasting plasma glucose; LDL-C, low-density lipoprotein cholesterol; HDL-C, high-density lipoprotein cholesterol; Pro-BNP, pro-brain natriuretic peptide; HbA1c, glycosylated hemoglobin A1c; LVEF, left ventricular ejection fraction.

**Table 2:** Baseline characteristics of population stratified by TyG>9.19.

Characteristics	Total population (N=813)	TyG ≤ 9.19 (N=271)	TyG>9.19 (N=542)	P value
Demographics				
Age (years)	59.27 (51.87, 66.53)	59 (52, 67)	59 (52, 66)	0.866
Male, n(%)	598 (73.6%)	204 (25.1%)	394 (48.5%)	0.431
DBP (mmHg)	80 (70, 86)	80 (70, 85)	80 (70, 87)	0.594
SBP (mmHg)	130 (120, 145)	130 (120, 144.75)	130 (120, 145)	0.094
HR (bpm)	75 (68, 81)	74 (66, 80)	76 (68, 82)	0.055
Case history (n, %)				
Smoking	436 (53.6%)	158 (19.4%)	278 (34.2%)	0.059
Drinking	233 (28.7%)	82 (10.1%)	151 (18.6%)	0.476
T2DM	239 (29.4%)	27 (3.3%)	212 (26.1%)	<0.001
Hypertension	507 (62.4%)	155 (19.1%)	352 (43.3%)	0.032
previous MI	70 (8.6%)	23 (2.8%)	47 (5.8%)	0.930
Dyslipidemia	183 (22.5%)	44 (5.4%)	139 (17.1%)	0.002
Clinical diagnosis (n, %)				
UA	515 (63.3%)	170 (20.9%)	345 (42.4%)	0.797
NSTEMI	32 (3.9%)	8 (1%)	24 (3%)	0.308
STEMI	237 (29.2%)	83 (10.2%)	154 (18.9%)	0.513
Coronary angiography (n, %)				
Diffuse lesions	487 (59.9%)	168 (20.7%)	319 (39.2%)	0.390
Chronic total occlusions	227 (27.9%)	76 (9.3%)	151 (18.6%)	0.956
Multi-vessel disease	409 (50.3%)	132 (16.2%)	277 (34.1%)	0.519
Number of stents	2 (1,3)	2 (1,3)	2(1,3)	0.111
Medications at discharge (n, %)				
Aspirin	811 (99.8%)	271 (33.3%)	540 (66.4%)	0.802
Clopidogrel	726 (89.3%)	245 (30.1%)	481 (59.2%)	0.470
Ticagrelor	89 (10.9%)	28 (3.4%)	61 (7.5%)	0.691
ACEI/ARB	691 (85%)	228 (28%)	463 (56.9%)	0.627
β-blockers	670 (82.4%)	224 (27.6%)	446 (54.9%)	0.896
Insulin	71 (8.7%)	7 (0.9%)	64 (7.9%)	<0.001
Laboratory measurements				
WBC (10^9/L)	6.93 (5.73, 8.57)	6.97 (5.73, 8.59)	6.92 (5.73, 8.51)	0.954
Hb (g/L)	144 (132, 153)	145 (132.5, 155)	144 (131, 153)	0.256
PLT (10^9/L)	200 (160, 238)	200 (162, 244.5)	200 (159, 236.8)	0.343
Creatinine (umol/L)	66 (56.38, 77.7)	67.7 (58.75, 78)	65 (55.53, 77.4)	0.051
TC (mmol/L)	4.24 (3.69, 4.82)	4.12 (3.645, 4.66)	4.3 (3.7, 4.93)	0.008
TG (mmol/L)	2.27 (1.93, 2.98)	1.93 (1.8, 2.15)	2.64 (2.13, 3.44)	<0.001
FPG (mmol/L)	6.09 (4.93, 8.25)	4.84 (4.38, 5.40)	7.21 (5.75, 9.97)	<0.001
TyG index	9.40 (9.09, 9.78)	8.97 (8.85, 9.08)	9.63 (9.40, 9.99)	<0.001
LDL-C (mmol/L)	2.49 (2.01, 2.99)	2.47 (2.02, 2.91)	2.5 (2.01, 3.08)	0.599
HDL-C (mmol/L)	0.9 (0.77, 1.04)	0.93 (0.785, 1.08)	0.89 (0.77, 1.02)	0.005
Pro-BNP (ng/L)	193.9 (72.74, 600.62)	191.8 (66.30, 619.53)	195.15 (75.19, 582.62)	0.948
HbA1C (%)	5.8 (5.4, 6.78)	5.6 (5.3, 5.8)	6.1 (5.5, 7.3)	<0.001
LVEF (%)	64 (56, 69)	64 (56, 69)	64 (56, 69)	0.794

Abbreviations as in **Table 1**.

### Association between the TyG index and endpoint events

During the maximum follow-up period of 66 months, 100 (12.3%) patients developed MACCE, including 30 (3.7%) all-cause deaths, 12 (1.5%) nonfatal myocardial infarction, 8 (1.0%) nonfatal strokes, and 50 (6.2%) unplanned repeat revascularizations. Compared with those in the low TyG index group, the rates of MACCE (14.9% vs. 7.0%, P < 0.001), MACE (13.5% vs. 5.9%, P < 0.001), all-cause death (4.8% vs. 1.5%, P = 0.018), and unplanned repeat revascularization (7.4% vs. 3.7%, P = 0.039) were significantly higher in the high TyG index group. However, the rates of nonfatal stroke were similar between the two groups (**Table 3**).

**Table 3:** Comparison of endpoint events stratified by the optimal cut-off value of TyG index.

Variable, n (%)	Total population (N=813)	TyG ≤ 9.19 (N=271)	TyG>9.19 (N=542)	P value
MACCE	100 (12.3%)	19 (7.0%)	81 (14.9%)	0.001
MACE	89 (10.9%)	16 (5.9%)	73 (13.5%)	0.001
Nonfatal Stroke	8 (1.0%)	1 (0.3%)	7 (1.3%)	0.379
All-cause death	30 (3.7%)	4 (1.5%)	26 (4.8%)	0.018
Unplanned repeat revascularization	50 (6.2%)	10 (3.7%)	40 (7.4%)	0.039

TyG, triglyceride-glucose; MACCE, major adverse cardiovascular and cerebrovascular events; MACE, major adverse cardiovascular events.

**Figure 2:**
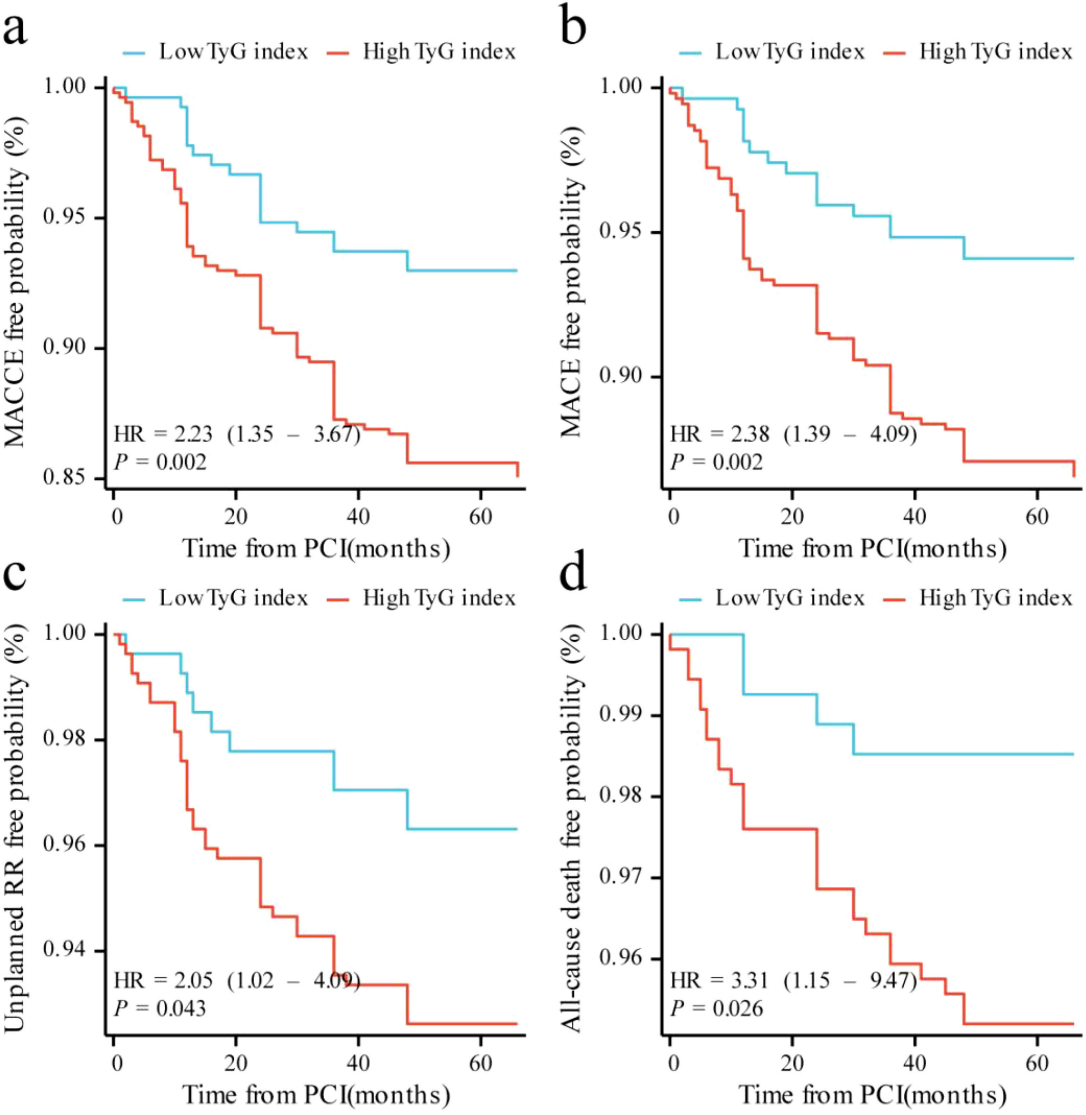
Kaplan–Meier curves for different endpoints: **(a)** MACCE; **(b)** MACE; **(c)** unplanned repeat revascularization; **(d)** all-cause death. MACCE, major adverse cardiac and cerebrovascular events; MACE, major adverse cardiovascular events; RR, repeat revascularization.

Kaplan–Meier curves showed that individuals with a high TyG index were more likely to develop MACCE (log-rank P = 0.002) and MACE (log-rank P = 0.002) than the patients in the low TyG index group (**Figure 2**). This increased incidence was primarily attributable to the growing risk of all-cause mortality (log-rank P = 0.026). Additionally, patients in the high TyG index group were more likely to experience unplanned repeat revascularization than those in the low TyG index group (log-rank P = 0.043).

**Figure 3:**
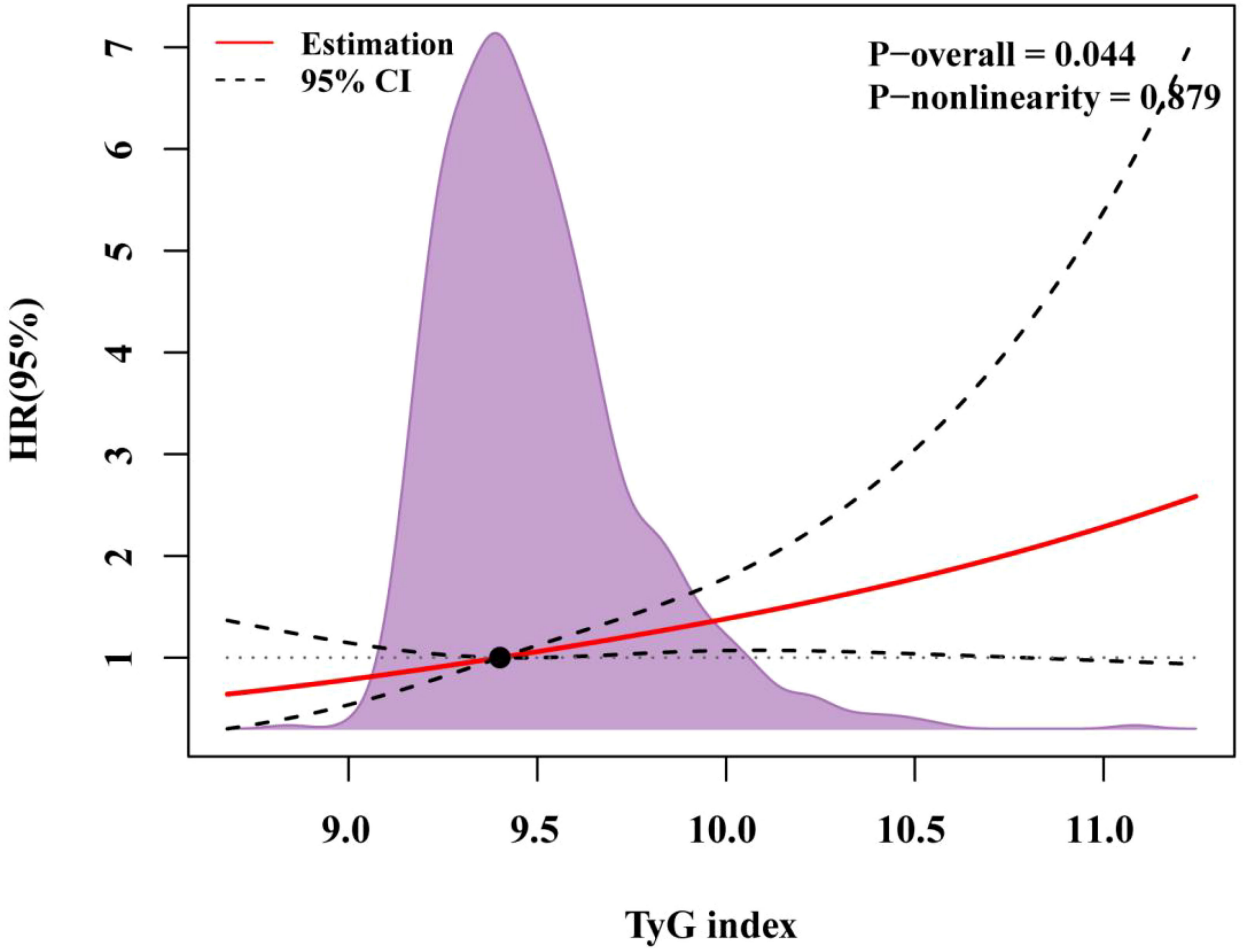
RCS regression for the adjusted dose–response relationship between the TyG index and MACCE. Data of the TyG index for repeat revascularization was fitted with a linear regression model using restricted cubic spines with three knots at the 5th, 50th, and 95th percentiles of the TyG index. Y-axis represents the hazardous ratio, and the dashed lines are 95% confidence intervals. OR, odds ratio.

**Table 4** presents the predictive value of TyG index for MACCE in different Cox proportional hazards models. Variables were chosen because they included statistically significant factors (P < 0.1) in univariate Cox regression analyses (**Supplementary Table S1**), as well as factors that might influence clinical outcomes. The TyG index was significantly associated with MACCE before adjustments for any variable (HR, 1.53; 95% CI 1.15–2.04; P = 0.004), with the high TyG index group at elevated risk (HR, 2.23; 95% CI 1.35–3.67; P = 0.002). After adjustment for general information, history, diagnosis (including age, sex, SBP, DBP, smoking, drinking, hypertension, previous MI, dyslipidemia, UA, NSTEMI, and STEMI) in Model 2, the TyG index remained an independent predictor for MACCE (HR, 1.41; 95% CI 1.03–1.95; P = 0.034). After adjustment for variables in Model 2 and key serologic test results (including WBC, Hb, PLT, LVEF, creatinine, TG, LDL-C, and HDL-C) in Model 3, a high TyG index remained significant for the occurrence of MACCE events (HR, 2.01; 95% CI 1.17–3.48; P = 0.012). After adjustment for variables in Model 3, procedure diagnoses and postoperative medications (including diffuse lesions, chronic total occlusions, three-vessel disease, number of stents, aspirin, clopidogrel, ticagrelor, ACEI/ARB, β-blockers, and insulin) in Model 4, the risk of MACCE in the high TyG group was 2.43 times higher than in patients with a TyG index < 9.19 (Model 4: HR, 2.43; 95% CI 1.37–4.31; P = 0.002). With the adjustment in Model 4, restricted cubic spline analysis depicted the relation between the TyG index and MACCE risk (**Figure 3**; P for nonlinearity = 0.879, P for overall trend = 0.044).

**Table 4:** Cox proportional hazards regression analysis.

	TyG index as a continuous variable			TyG index as a categorical variable		
	HR	95%CI	P value	HR	95%CI	P value
**Model 1**	1.53	1.15, 2.04	0.004	2.23	1.35, 3.67	0.002
**Model 2**	1.41	1.03, 1.95	0.034	2.25	1.33, 3.79	0.002
**Model 3**	1.59	1.05, 2.40	0.028	2.01	1.17, 3.48	0.012
**Model 4**	1.72	1.13, 2.63	0.012	2.43	1.37, 4.31	0.002

Model 1: no covariates were adjusted;

Model 2: adjusted for age, gender, SBP, DBP, smoking, drinking, hypertension, OMI, dyslipidemia, UA, NSTEMI, STEMI;

Model 3: adjusted for variables in Model2 and WBC, Hb, PLT, LVEF, creatinine, TG, LDL-C, HDL-C;

Model 4: adjusted for variables in Model3 and diffuse lesions, Chronic total occlusions, three-vessel disease, Number of stents, Aspirin, Clopidogrel, Ticagrelor, ACEI/ARB, b-blockers, insulin..

a The HR was examined by the increase of each unit in TyG index

b The HR was examined referring the low TyG index group

HR, hazard ratio; CI, confidence interval; other abbreviations as in **Table 1**.

### Subgroup analysis

**Figure 4:**
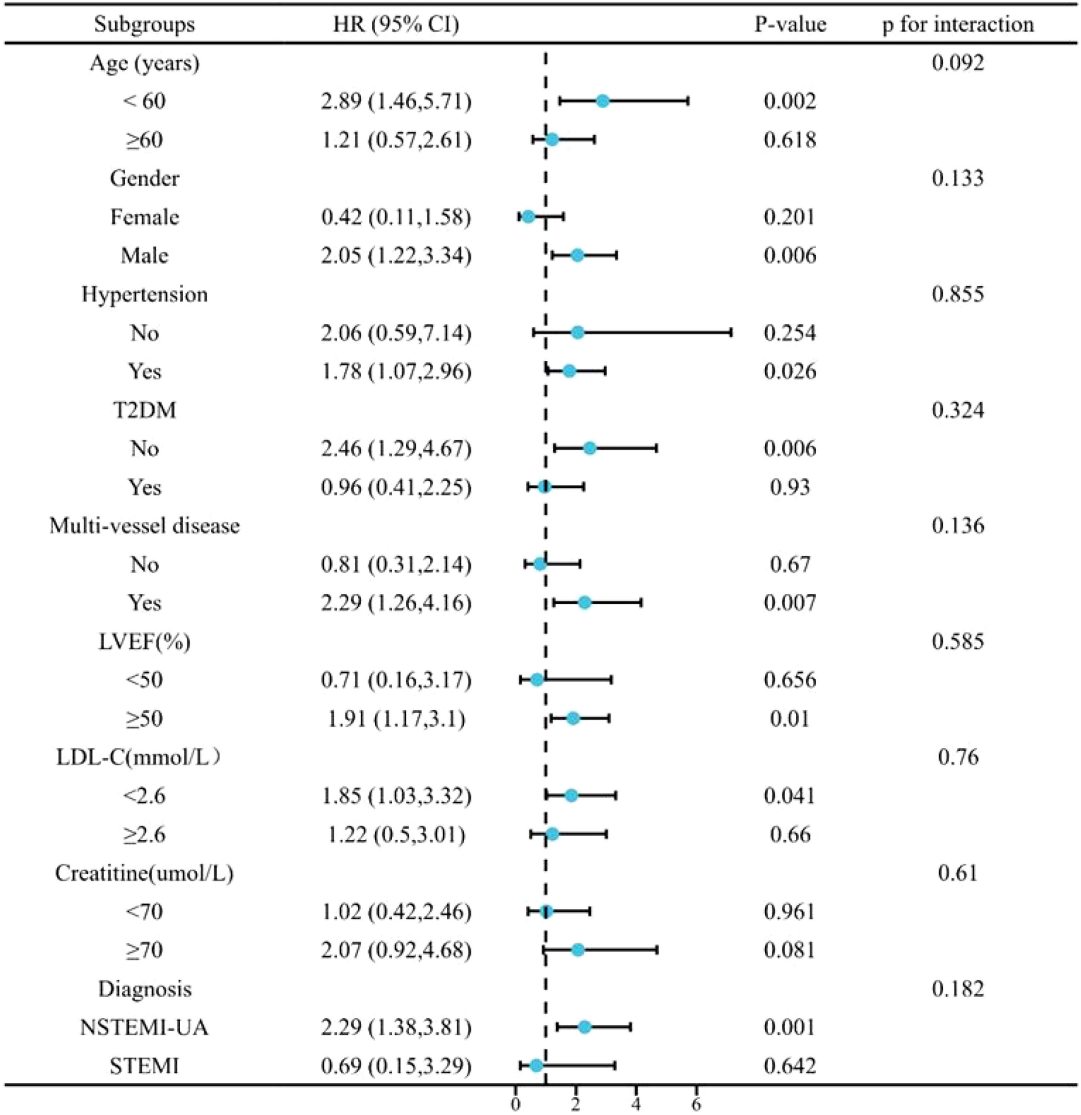
Subgroup analysis for the impact of the TyG index on MACCE. MACCE, major adverse cardiac and cerebrovascular events; TyG, triglyceride-glucose.

Subgroup analysis evaluated the influence of the TyG index as a continuous variable on MACCE in Model 4 based on age, sex, hypertension, T2DM, LDL-C, LVEF, and creatinine levels. As shown in **Figure 4**, no significant interaction was observed among the subgroups (P > 0.05). However, it was observed that in older men with hypertension and multivessel CAD (indicated by coronary angiography), LVEF ≥ 50%, LDL-C < 2.6 mmol/L, and creatinine ≥ 70 µmol/L, the incidence of MACCE was more likely to increase (P < 0.1). In patients undergoing elective PCI surgery, which were diagnosed with NSTEMI or UA, the incidence of MACCE was more likely to increase with the increase of TyG index (P < 0.1).

## Discussion

In our retrospective study, we identified a correlation between the TyG index and MACCE in patients with hypertriglyceridemia undergoing DES-PCI. The major findings were as follows: (1) the TyG index could independently predict MACCE, with its significance remaining after adjusting for all covariates; (2) the risk of MACCE increased with an increase in the TyG index; and (3) patients with a TyG index > 9.19 exhibited higher occurrences of MACCE, MACE, unplanned repeat revascularization, and all-cause death compared with those of patients with a TyG index < 9.19. Our findings suggest the potential value of the TyG index in patients with hypertriglyceridemia who underwent DES-PCI.

The relation between hypercholesterolemia and atherosclerosis is well established, and LDL­C lowering therapy serve as the foundation for reducing the risk of atherosclerotic cardiovascular disease (11). However, even with adequate LDL-C control, a significant residual cardiovascular risk remains, with hypertriglyceridemia being one such risk factor (12). Plasma TG levels fluctuate considerably with daily meals. Individuals with underlying lipid or glucose metabolism dysfunction, such as T2DM or obesity, tend to experience prolonged hypertriglyceridemia compared with those without metabolic dysfunction (13). Long-term hypertriglyceridemia can lead to multiple metabolic abnormalities, including hyperglycemia, oxidative stress, and elevated levels of coagulation factors (14).

The TyG index, which incorporates blood glucose and TG levels, is a simple and convenient method for evaluating the metabolic status. As a systemic metabolic dysfunction, IR is considered significantly related to CVD and T2DM (15). Compared to the HIEC and HOMA-IR methods, the TyG index is easier to access through a single blood sample. Zou analyzed the TyG index in different obesity phenotypes and normal body mass index (BMI), categories and demonstrated the prognostic value of the TyG index in metabolic dysfunction (16). Additionally, the concept of cardiovascular-kidney-metabolic syndrome has recently been proposed because of the interplay between metabolic, renal, and cardiovascular diseases (17). Recent prospective studies have indicated that the TyG index can independently predict the incidence of chronic kidney disease in a non-diabetic population (18). By comparing various factors associated with MACCE in patients with CVD after PCI, one study identified the TyG index as a non-insulin-based marker for risk assessment (9). Thus, the TyG index serves as a surrogate marker of metabolic status and may predict outcomes in the CVD population.

Although the TyG index has demonstrated good predictive efficacy in different populations, few studies have focused on patients with hypertriglyceridemia undergoing DES-PCI. In our study, we enrolled 813 patients with elevated TG levels who underwent coronary stent implantation. Patients who experienced MACCE had high TyG and HbA1c levels (**Table 1**). The baseline TyG index, stratified by a cutoff of 9.19, indicated that patients with a high TyG index were more susceptible to hypertension and diabetes (**Table 2**). When comparing the TyG index to FPG or HbA1c in predicting MACCE previous studies have suggested that it is more predictive of cardiovascular events in patients with ACS (19). However, these conclusions remain controversial. The ROC curve showed no distinct differences in the predictive effect of the TyG index and HbA1c, LDL-C, or BNP (**Supplementary Table S1**). These differences may be attributed to the effects of hypoglycemic and lipid-lowering treatments. Additionally, some indicators may have been influenced by non-fasting conditions.

ROC analysis indicated that the cutoff value for predicting MACCE in our patients with hypertriglyceridemia was 9.19. This cutoff value also had predictive value for endpoints, such as MACE, unplanned repeat revascularization, and all-cause death (P < 0.05). Differences in ethnic groups and clinical characteristics may account for variations in the TyG index cutoff values. In previous studies, the cutoff value of the TyG index based on the ROC method ranged between 8.5 and 9 (20). Because our population consists of patients with high TG levels, the cutoff value of the TyG index was higher than that of the general population. In the baseline dataset (**Table 2**), we observed that patients with a TyG index > 9.19 had higher systolic blood pressure and heart rates, as well as more frequent histories of smoking, comorbid hypertension, or diabetes, than those of patients with a TyG index < 9.19. Regarding medications, more patients received insulin treatment. As a high TyG index reflects the state of IR, it provides a reference for selecting follow-up treatments (including insulin sensitizers) (21).

The TyG index was correlated with the risk of MACCE, and nonlinearity could not be proved (nonlinear P = 0.879, P overall = 0.044), which is consistent with published research (22). An observational study of post-PCI patients with T2DM and non-ST-segment elevation myocardial infarction found that an increase in the TyG index elevated the risk of MACCE, independent of other risk factors (23). Similar findings have been observed in patients with chronic coronary syndromes, regardless of whether they had T2DM (24, 25). These results suggest that even small increases in the TyG index can be clinically significant, emphasizing the importance of monitoring and managing TyG levels in patients to mitigate MACCE risk.

The subgroup analysis in **Figure 4** showed that patients with non-T2DM, who may not be suitable for insulin-based measures, had a higher risk of MACCE events with a high TyG index. A high TyG index was also associated with the incidence of MACCE incident in men (< 60 years old), patients with hypertension, non-T2DM, multivessel-CAD, LVEF ≥ 50%, LDL < 2.6 mmol/L, or creatinine ≥ 70 µmol/L (P < 0.1). Recent findings have highlighted the predictive value of the TyG index in chronic kidney disease (26). TyG monitoring warrants further surveillance and intervention in patients with hypertriglyceridemia with those typical.

These findings may aid in identifying high-risk patients, optimizing secondary prevention, and adjusting therapeutic approaches for patients with CVD and lipid metabolism disorders. Further research is needed to verify our findings and uncover the underlying mechanisms between TGs and cardiovascular disease.

### Study limitations

This study had some limitations. First, it was a single-center retrospective study based on a relatively small sample size, which was the first to apply the TyG index to the prognosis of patients with hypertriglyceridemia undergoing DES-PCI. Second, the TyG index was calculated on admission, and follow-up examinations require further evaluation. Plasma TG levels may also be affected by other treatments. Moreover, the study lacked relevant data on other indicators of metabolic disorders, such as waist circumference, BMI. Further research on the longitudinal TyG fluctuations due to the effects of different drugs is ongoing. Finally, our results need to be verified in multicenter prospective cohort studies.

## Conclusions

Our study is the first to apply the TyG index to patients with hypertriglyceridemia undergoing DES-PCI. In this population, an increase in the TyG index was associated with an elevated risk of MACCE after adjusting for other variables. Patients with a high TyG index (> 9.19) had a higher risk of MACE, unplanned repeat revascularization, and all-cause death than that of patients with a low TyG index.

## Data Availability

The raw data supporting the conclusions of this article will be made available by the authors, without undue reservation.
