# Supplementary material for: Association between high triglyceride-glucose index and MACCE in hypertriglyceridemia patients undergoing percutaneous coronary intervention
Source: Front Endocrinol (Lausanne). 2025 Apr 14;16:1519895. doi: 10.3389/fendo.2025.1519895 (PMC12034535; doi:10.3389/fendo.2025.1519895)

**Table S1. Cox proportional hazards Regression analyze**

|  | Univariate | | Multivariate | |
| --- | --- | --- | --- | --- |
|  | HR (95% CI) | P-value | Adjusted HR (95% CI) | P-value |
| Demographics | | | | |
| Age (years) | 1.02 (1.00, 1.04) | 0.024 | 1.01 (0.99, 1.03) | 0.483 |
| Male, n (%) | 0.93 (0.60, 1.43) | 0.727 |  |  |
| SBP (mmHg) | 1.00 (0.99, 1.01) | 0.555 |  |  |
| DBP (mmHg) | 0.99 (0.98, 1.01) | 0.500 |  |  |
| HR (bpm) | 1.02 (1.00, 1.03) | 0.040 | 1.01 (0.99, 1.03) | 0.301 |
| Medical history (n, %) | | | | |
| Smoking | 1.06 (0.72, 1.57) | 0.769 |  |  |
| Drinking | 0.77 (0.49, 1.22) | 0.271 |  |  |
| Diabetes | 1.59 (1.06, 2.38) | 0.024 | 1.42 (0.82, 2.45) | 0.212 |
| Hypertension | 1.31 (0.86, 1.99) | 0.212 |  |  |
| OMI | 1.85 (1.05, 3.25) | 0.033 | 1.50 (0.78, 2.88) | 0.227 |
| Dyslipidemia | 0.80 (0.49, 1.32) | 0.391 |  |  |
| Clinical diagnosis | | | | |
| UA, n (%) | 1.13 (0.75, 1.71) | 0.562 |  |  |
| NSTEMI, n (%) | 0.49 (0.12, 1.99) | 0.320 |  |  |
| STEMI, n (%) | 0.80 (0.51, 1.25) | 0.324 |  |  |
| Coronary angiography | | | | |
| Diffuse lesions | 1.26 (0.84, 1.90) | 0.271 |  |  |
| Chronic total occlusions | 1.08 (0.70, 1.66) | 0.734 |  |  |
| Multi-vessel disease | 1.75 (1.16, 2.62) | 0.007 | 1.77 (1.11, 2.82) | 0.016 |
| Number of stents | 1.04 (0.86, 1.25) | 0.675 |  |  |
| Medications at discharge (n, %) | | | | |
| Aspirin | 0.13 (0.02, 0.95) | 0.044 | 0.13 (0.02, 1.02) | 0.052 |
| Clopidogrel | 0.96 (0.51, 1.79) | 0.886 |  |  |
| Ticagrelor | 1.25 (0.70, 2.24) | 0.451 |  |  |
| ACEI/ARB | 1.46 (0.78, 2.73) | 0.238 |  |  |
| b-blockers | 1.78 (0.95, 3.34) | 0.070 | 1.50 (0.74, 3.04) | 0.257 |
| Insulin | 1.78 (1.01, 3.13) | 0.046 | 1.03 (0.51, 2.09) | 0.929 |
| Laboratory measurements | | | | |
| WBC (10^9/L) | 0.97 (0.89, 1.06) | 0.481 |  |  |
| Hb (g/L) | 0.99 (0.98, 1.00) | 0.022 | 0.99 (0.98, 1.00) | 0.197 |
| PLT (10^9/L) | 1.00 (1.00, 1.00) | 0.290 |  |  |
| Creatinine (ummol/L) | 1.00 (1.00, 1.00) | 0.248 |  |  |
| TC, (mmol/L) | 1.01 (0.94, 1.07) | 0.866 |  |  |
| TG, (mmol/L) | 1.01 (0.98, 1.04) | 0.695 |  |  |
| FPG, (mmol/L) | 1.04 (1.00, 1.09) | 0.060 | 0.96 (0.89, 1.04) | 0.283 |
| TyG index | 1.53 (1.15, 2.04) | 0.004 | 1.60 (1.06, 2.42) | 0.026 |
| LDL-C (mmol/L) | 1.01 (1.00, 1.01) | 0.030 | 1.00 (0.94, 1.05) | 0.871 |
| HDL-C (mmol/L) | 0.87 (0.33, 2.31) | 0.781 |  |  |
| Pro-BNP (ng/L) | 1.00 (1.00, 1.00) | <0.001 | 1.00 (1.00, 1.00) | 0.013 |
| HbA1C (%) | 1.03 (0.99, 1.08) | 0.179 |  |  |
| LVEF (%) | 0.97 (0.96, 0.99) | <0.001 | 0.99 (0.97, 1.00) | 0.103 |

**Table S2. ROC result table**

| Variants | Time(years) | Cut-off value | PPV | NPV | sensitivities | specificities |
| --- | --- | --- | --- | --- | --- | --- |
| TyG | 3 | 9.194 | 0.795 | 0.649 | 0.128 | 0.935 |
| TG | 3 | 2.560 | 0.513 | 0.357 | 0.147 | 0.917 |
| FPG | 3 | 7.340 | 0.462 | 0.317 | 0.148 | 0.914 |
| LDL-C | 3 | 3.430 | 0.205 | 0.130 | 0.158 | 0.901 |
| Pro-BNP | 3 | 1897.000 | 0.154 | 0.066 | 0.218 | 0.902 |

**Table S3. AUC information table.**

| Variants | Time(years) | **AUC** | **95%CI** | **cumulative survival rate** | **cumulative incidence** |
| --- | --- | --- | --- | --- | --- |
| TyG | 3 | 0.584 | 0.5161 - 0.6509 | 0.893 | 0.107 |
| TG | 3 | 0.576 | 0.511 - 0.6419 | 0.893 | 0.107 |
| FPG | 3 | 0.575 | 0.5036 - 0.646 | 0.893 | 0.107 |
| LDL-C | 3 | 0.515 | 0.442 - 0.5873 | 0.893 | 0.107 |
| Pro-BNP | 3 | 0.535 | 0.4664 - 0.6042 | 0.893 | 0.107 |

**Table S4. Comparison of endpoint events stratified by the optimal cut-off value of TyG index**

| Variable, n(%) | Total  (N = 2186) | TG < 1.7  (N = 1373) | TG ≥ 1.7  (N = 813) | P value |
| --- | --- | --- | --- | --- |
| MACCE | 300 (13.7%) | 200 (14.6%) | 100 (12.3%) | 0.137 |
| MACE | 270 (12.4%) | 180 (13.1%) | 90 (11.1%) | 0.161 |
| Nonfatal Stroke | 25 (1.1%) | 17 (1.2%) | 8 (1%) | 0.589 |
| All-cause death | 105 (4.8%) | 75 (5.5%) | 30 (3.7%) | 0.061 |
| Unplanned repeat revascularization | 127 (5.8%) | 77 (5.6%) | 50 (6.15%) | 0.601 |

TyG, triglyceride-glucose; MACCE, major adverse cardiovascular and cerebrovascular events; MACE, major adverse cardiovascular events

**Figure. S1 ROC analysis of the TyG index, LDL-C,** **Pro-BNP, TG and FBG to predict MACCE.** AUC, area under the curve; TyG, triglyceride-glucose; other abbreviations as in Table 1
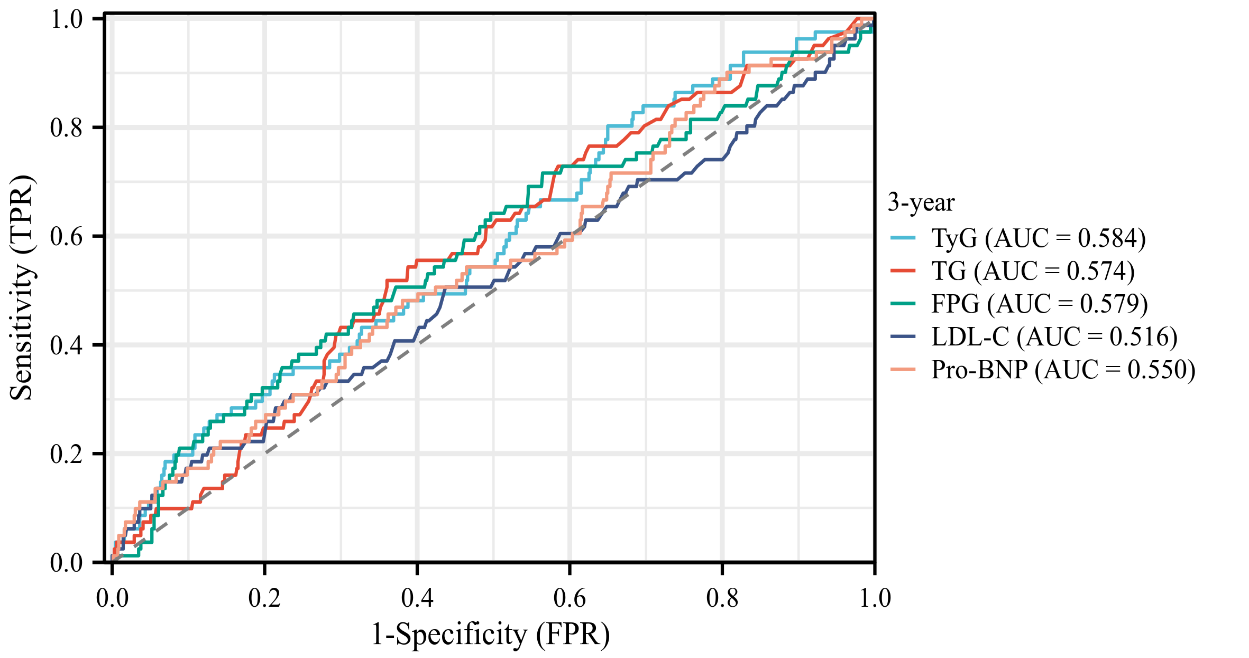


**Figure. S2 AUC analysis of the TyG index, LDL-C, Pro-BNP, TG and FBG to predict MACCE.** AUC, area under the curve; TyG, triglyceride-glucose; other abbreviations as in Table 1


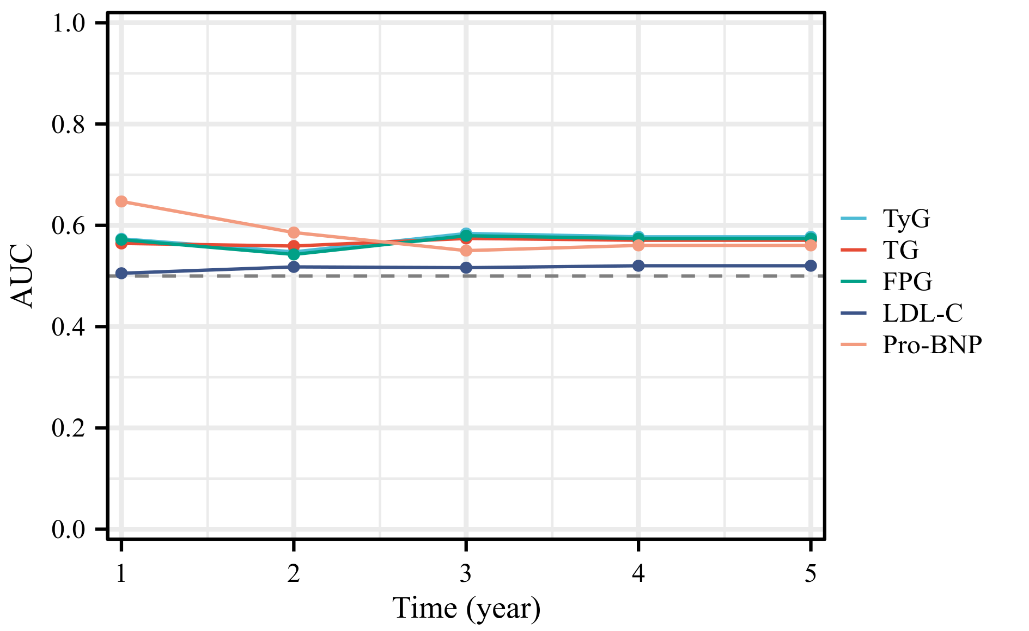


**Figure. S3 RCS analysis of different endpoints.** a MACCE; b MACE, c All-cause death, d. Unplanned repeat revascularization


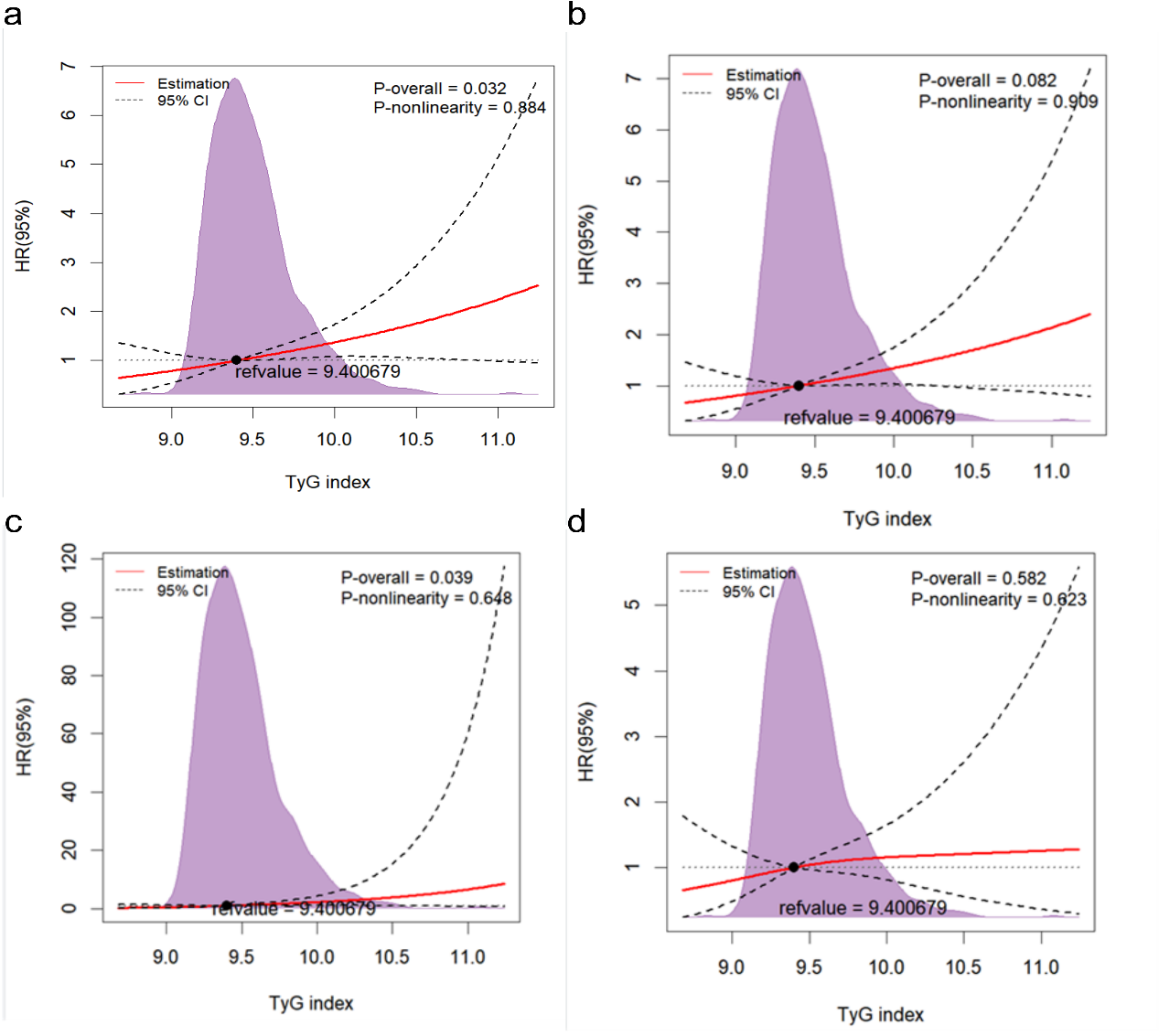

Supplement: Supplementary file 1 [file DataSheet1.docx]
